# Evaluation of the Efficacy of Fludora^®^ Fusion WP-SB 56.25 (Mixture of Clothianidin and Deltamethrin) against *Anopheles coluzzii* Laboratory and *An. arabiensis* Wild Colonies

**DOI:** 10.3390/tropicalmed7100316

**Published:** 2022-10-19

**Authors:** Marième Gueye, Ibrahima Dia, Seynabou Diedhiou, Badara Samb, Abdoulaye Kane Dia, Moussa Diagne, Ousmane Faye, Lassana Konaté

**Affiliations:** 1 Laboratoire d’Ecologie Vectorielle et Parasitaire (LEVP), Faculté des Sciences et Techniques, Université Cheikh Anta Diop de Dakar, Dakar BP 5005, Senegal; 2 Pôle de Zoologie Médicale, Institut Pasteur de Dakar (IPD), Dakar BP 220, Senegal; 3 Service de Lutte Anti Parasitaire (SLAP) de Thiès, Ministère de la Santé et de l’Action Sociale, Dakar BP 4024, Senegal

**Keywords:** *Anopheles*, Fludora^®^ Fusion, clothianidin, deltamethrin, hut, efficacy, Senegal

## Abstract

For malaria control, the application of long-lasting insecticidal nets and indoor residual spraying has led to a significant reduction in morbidity and mortality. However, the sustainability of these gains is hampered by the increase in insecticide resistance. It is therefore judicious to evaluate new insecticide formulations. In comparison to clothianidin and deltamethrin, the efficacy and residual effect of Fludora^®^ Fusion was evaluated using an *Anopheles coluzzii* laboratory and *An. arabiensis* wild colonies in huts from August 2016 to June 2017 on cement and mud walls. Mortality was recorded at 24, 48, 72, and 96 h post exposure. Like deltamethrin and clothianidin, Fludora^®^ Fusion showed delayed mortality rates above the WHO’s 80% threshold over a period of 11 months with the laboratory strain. With the wild strain, while residual efficacy was observed at 2 months for the three insecticides, no residual efficacy was observed at 8 months at 24 h in both substrates. However, the increased efficacy was observed with increased holding periods (72 h and 96 h). These findings suggest that Fludora^®^ Fusion could be an alternative candidate since this duration covers the transmission period in most areas in Senegal.

## 1. Introduction

Despite the significant decline in morbidity and mortality observed in recent years, malaria is still one of the major public health problems in sub-Saharan Africa [1,2]. The scaling-up of insecticide-based strategies such as long-lasting insecticidal nets (LLINs) and indoor residual spraying (IRS) is still considered effective in preventing the transmission of this disease. For the specific case of IRS, despite its ability to reduce transmission, huge variations in the duration of efficacy have been observed depending on the insecticide use [3,4,5]. The widespread occurrence of pyrethroid resistance in most malaria-endemic countries in Africa, and the emergence of resistance to carbamates, raise many concerns. Thus, preserving the sustainability of this strategy represents a major challenge for vector control. An alternative proposed to prevent the rapid and widespread resistance is the rotational use of two or three different insecticides [6]. However, due to the scarcity of alternative insecticides and the existence of cross-resistance between some of them, preserving the residual efficacy of the existing tools such as IRS requires the development of new innovative tools. The WHO recommended for IRS the rotational use of different classes of insecticides with different modes of action, and the development of mixtures or combinations for resistance management. However, whatever the combination of such insecticides for resistance management in mosquitoes, an evaluation of its residual efficacy and its persistence on the treated surfaces is essential before any generalized use. Fludora^®^ Fusion, a combination of the neonicotinoid clothianidin and the pyrethroid deltamethrin formulation developed by Bayer CropScience, was proposed for IRS. The targets of clothianidin in the central nervous system of insects are nicotinic acetylcholine receptors. They cause overstimulation that can lead to paralysis and death of the insect [7]. For pyrethroids, the mechanism is different. These insecticides are neurotoxic as they interact with the voltage-gated sodium channels in the neurons of insects. Thus, formulations targeting clothianidin can be included in the malaria vector control toolbox, especially in areas with high levels of resistance to multiple insecticide classes [8,9]. Indeed, the greatest benefit is achieved when the target mosquito population is fully susceptible to the constituents of the mixture [10].

In Senegal, the National Malaria Control Program (NMCP) in collaboration with the President’s Malaria Initiative (PMI), launched the IRS program in 2007 for the control of malaria vectors. Between 2007 and 2009, pyrethroid-based insecticides were used: ICON WP 10 (lambdacyhalothrin) from 2007 to 2009, and the K-Othrine WG 250 (deltamethrin) from 2010 to 2011 [11]. Due to the appearance of resistance phenomena after five years of use to both insecticides [11], this class of insecticide was replaced by bendiocarb (FICAM^®^ WP 80) and 300 CS formulation of Actellic. Although the residual efficacy of 3–6 months was observed with FICAM^®^ WP 80 [12], there was an urgent need to search for durable and cost-effective products for IRS interventions.

An evaluation of the efficacy and residual effect of Fludora^®^ Fusion on a wild *An. arabiensis* and laboratory *An. coluzzii* colonies was carried out during this work with the final aim of using it for malaria vector control.

## 2. Materials and Methods

### 2.1. Study Sites

The study was undertaken in an experimental hut station located about 400 m from the edge of Ndioukhane village (14°42′13.05 N, 16°50′54.88 W) situated about 12 km from the city of Thiès, and 70 km from Dakar, the capital of Senegal.

The experimental station is located at about half a kilometer from the market-garden wells near the village, which are breeding sites for *Anopheles* mosquitoes. Ten huts were built according to the model used in West Africa [13,14]. The distance between two huts is 5.3 m. Each hut is 2.64 m, 2 m, and 2 m in length, width, and height, respectively, and is built from cement bricks. The inner wall and the floor are covered with cement or mud in 6 and 4 huts, respectively. The roof is covered on the outside with corrugated iron and on the inside with a plywood ceiling. Each hut has four entrances designed to allow mosquitos to enter but prevent them from escaping once they have entered the hut; a fully screened veranda trap equivalent to an exit is located on the 4th side and collects exophilic mosquitoes.

### 2.2. Insecticide Formulations Used

- Fludora^®^ Fusion is an insecticide developed for IRS. The formulation type is a Wettable Powder (WP-SP) available in 100 g soluble sachets. It contains two active ingredients: 500 g/kg clothianidin and 62.5 g/kg deltamethrin. The application rate of the product is 200 mg of clothianidin/sqm and 25 mg of deltamethrin/sqm.
- K-Othrine WG 250: containing 250 g/kg deltamethrin applied at 25 mg deltamethrin/sqm. The product is diluted in 10 L of water and applied to an area of 250 sqm (i.e., 40 mL per sqm).
- Clothianidin WG70: applied at 200 mg clothianidin/sqm. One 100 g sachet is dissolved in 10 L of water to spray an area of 250 sqm.

### 2.3. Mosquito Colonies Used

A laboratory colony of *An. coluzzii* used in previous studies [12,15] and colonized in 2008 from specimens collected in the suburb of Essos in Yaounde, Cameroon was used. As a wild colony, *An. arabiensis* larvae were collected in the locality of Pikine (14°42′33 N, 17°23′59 W), a suburb of Dakar in October 2016 and April 2017. Both colonies were maintained until the adult stage under standard conditions (27 ± 2 °C; 75% ± 5% relative humidity with a photoperiod of 12 h during the day and 12 h at night).

### 2.4. Treatment of Huts

The efficacy and residual effect of Fludora^®^ Fusion on the cement and mud walls were studied and compared to clothianidin (WG70) alone and deltamethrin (WG250) alone. The treatments of the huts were carried out using the WHO recommended compression sprayer (10-litre Hudson Xpert^®^ 67422 AD pumps).

For each of the two products (Fludora^®^ Fusion and clothianidin), 2 huts with cement walls and 1 with mud walls were selected and treated; while for K-Othrine, 2 huts were selected and treated (1 with cement walls and 1 with mud walls). Two untreated huts (one with cement walls and one with mud walls) were selected as controls.

Two mosquito colonies were used: (i) a laboratory *An. coluzzii* colony on day 7 and then every month until month 11 after the treatments from August 2016 to June 2017; (ii) a local wild colony of *An. arabiensis*, 2 months after the treatment and after 8 months in the experimental station.

Before the insecticide treatment, the walls of the huts were completely cleaned, and the absence of insecticide was confirmed by cone bioassay tests using the *An. coluzzii* laboratory colony.

### 2.5. Insecticide Susceptibility Tests

The susceptibility of the *An. coluzzii* laboratory and *An. arabiensis* wild colonies to deltamethrin was assessed using standard WHO test kits for adult mosquitoes [16]. Tests were performed on unfed 3–5-day-old females, in batches of 20–25 females, exposed to insecticide-impregnated papers for 1 h. For each test, two batches of 20–25 females were exposed to untreated papers as the control. After 1 h exposure, female mosquitoes were kept at a temperature of 27 ± 2 °C and a relative humidity of 80 ± 10%, and the number of dead female mosquitoes was recorded 24 h after exposure.

### 2.6. Bioassay Tests

The efficacy of the insecticides was evaluated according to the World Health Organization’s standard cone test on wall surfaces [17,18]. In each of the treated and untreated huts, one cone was attached to each of the four walls. In each cone, ten unfed female mosquitoes aged between 2 to 5 days were introduced for a 30 min exposure. At the end of this exposure, the mosquitoes were transferred to cups labeled with the corresponding information from each cone per box. The cups were then stored at a temperature of 27 ± 2 °C and a relative humidity of 80 ± 10% and the mosquitoes were fed with a 10% sugar solution. The mortalities of mosquitoes were recorded 24 h after exposure from the 7th day to 3 months after the treatments. Because of the existence of surviving mosquitoes 24 h after the exposure, the mosquito mortality was recorded at 24 h and 48 h in the 4th month; then at 24 h, 48 h, and 72 h in the 5th month; and for the following months, at 24 h after exposure and daily until 96 h.

### 2.7. Data Analysis

The data were entered into an Excel Sheet and the analyses performed with R software (version 3.6.1). Treatment efficacy was assessed according to the WHO criteria to determine the residual treatment effect by comparing observed mortality rates to the 80% cut-off criteria [16]. For the susceptibility tests, the status was determined using the WHO criteria: mortality rates > 98% indicated susceptibility, whereas mortality rates between 90% and 97% indicated suspected resistance. Abbot’s formula was used to correct the mortality rates when the mortality rates in the control were between 5 and 20%.

## 3. Results

### 3.1. Insecticide Susceptibility Tests

The results of insecticide susceptibility testing of *An. coluzzii* laboratory and *An. arabiensis* wild colonies are presented in Table 1. A total of 106 and 111 individuals were exposed. Susceptibility to deltamethrin was observed for *An. coluzzii* (morality rate = 98.1%), while for *An. Arabiensis* the mortality was 11.9%. These results indicate that the *An. Arabiensis* was resistant, whereas *An. coluzzii* was fully susceptible.

### 3.2. Residual Efficacy of the Treatments against the An. coluzzii Laboratory Colony

**Figure 1:**
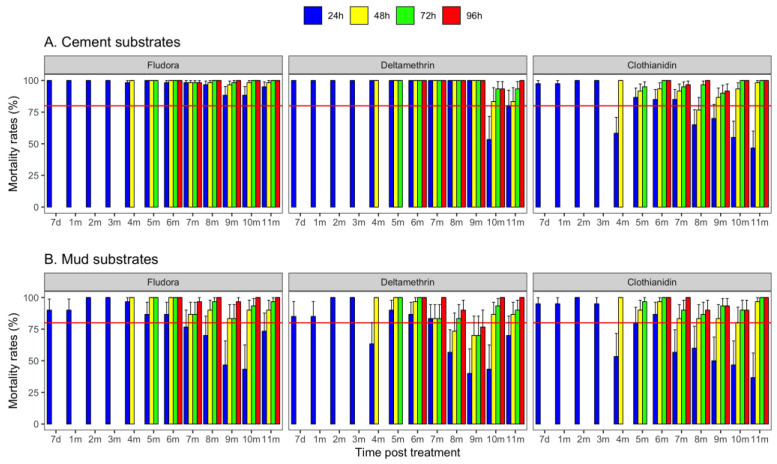
Mortality rates recorded after exposure of the *An. coluzzii* susceptible colony on cement (**A**) and mud (**B**) supports in the experimental station from the 7th day to the 11th month after the treatments with Fludora^®^ Fusion, deltamethrin, and clothianidin at 24 h, 48 h, 72 h, and 96 h. The horizontal red line indicates the 80% WHO threshold limit of efficacy. The bars indicate the upper limits of the 95% confidence intervals associated with the mortality rates.

The residual efficacy of Fludora^®^ Fusion using the laboratory colony *An. coluzzii* was better than deltamethrin and clothianidin on cement walls (Figure 1A). The mortality rates observed with Fludora^®^ Fusion exceeded the WHO efficacy threshold (80%) over the 11-month period for all the holding times, whereas for deltamethrin, the treatment was effective for the whole period except for the 10th month, during which the 24 h mortality rate was below the efficacy threshold (53.3%). Delayed mortalities at 48 h, 72 h, and 96 h were over 80% from the 4th month to the end of the 11-month study.

On mud surfaces, the 24 h mortality rates were above 80% up to 3 months for all three products (Figure 1B). For Fludora^®^ Fusion, the efficacy was prolonged until month 6 for all the holding times, and the product did not reach the 80% threshold until the end of the study at the holding period of 24 h; the same was observed for the other two products. The delayed mortalities at 48 h, 72 h, and 96 h were above 80% from month 6 to the end of the study with the exception of deltamethrin at month 9 (Figure 1B).

### 3.3. Residual Effect of the Treatments against the Wild An. arabiensis Colony

**Figure 2:**
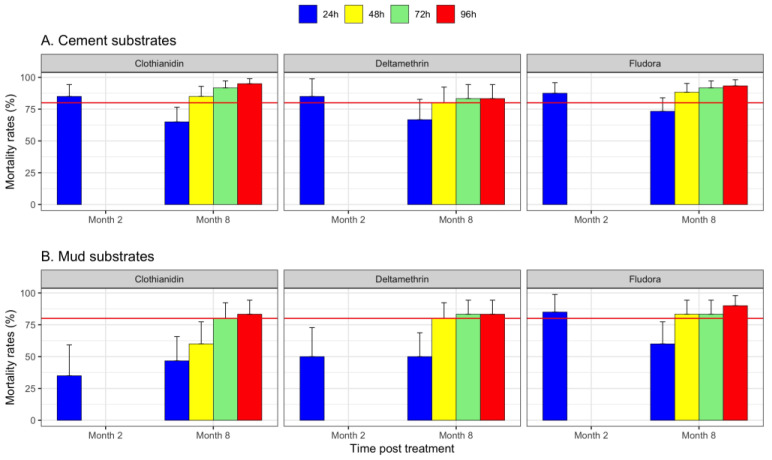
Mortality rates recorded after exposure of the *An. arabiensis* resistant strain on cement (**A**) and mud (**B**) supports in the experimental station, 2 and 8 months after the Fludora^®^ Fusion, deltamethrin, and clothianidin at 24 h, 48 h, 72 h, and 96 h. The horizontal red line indicates the 80% WHO threshold limit of efficacy. The bars indicate the upper limits of the 95% confidence intervals associated with the mortality rates.

Due to the lack of enough mosquitoes, the efficacy of the treatments was only carried out twice (2 months and 8 months after the treatments, respectively). This evaluation started in October 2016, two months after the treatments in August 2016. The immediate mortalities determined at 24 h after exposure were above the 80% efficacy threshold only on cement surfaces for all three products, and only for Fludora^®^ Fusion on the mud surfaces (Figure 2A).

In April 2017, 8 months after the treatments, the 24 h mortality was below 80% on both substrates for all three products (Figure 2A,B). At 48 h, 72 h, and 96 h, an effective residual effect on the cement and mud walls was observed for Fludora^®^ Fusion as well as for deltamethrin and clothianidin with the exception of clothianidin on mud surfaces at 48 h (mortality rate = 60%).

## 4. Discussion

The use and scaling-up of control tools in Senegal in recent years yielded encouraging results with a decrease in the prevalence of the malaria parasite in children under 5 years of age [19,20]. The national prevalence of malaria has been significantly reduced from 3% in 2012–2013 to 0.4% in 2017 with huge variations between ecological zones and sites [21]. Since 2007, as part of a large-scale program to implement and scale-up malaria control activities, the NMCP supported by the US President’s Malaria Initiative (PMI) has implemented IRS in pilot districts. Thus, from 2007 to 2009, pyrethroids, namely ICON WP 10 and K-Othrine WG 250, were successively used and lead to the emergence of resistance to these insecticides [11]. Thus, in the subsequent years, from 2011 to 2014, bendiocarb (FICAM WP 80) and Actellic 300CS were used to overcome pyrethroid resistance. While efficacy was observed, the residual effect did not exceed 3 to 6 months depending on the sites studied, suggesting at least two treatment cycles to cover the entire transmission season [12].

One of the main limitations of this study is the lack of quality control data on the treatments. However, the evaluation of the residual efficacy of the treatments 7 days after spraying using the laboratory colony showed on cement and mud supports that Fludora^®^ Fusion, deltamethrin, and clothianidin were effective.

The susceptibility tests confirmed that the laboratory colony *An. coluzzii* was fully susceptible to deltamethrin, while the *An. arabiensis* wild colony was resistant. Thus, the two colonies were appropriate to study the efficacy and residual effect of treatments.

Our results showed that Fludora^®^ Fusion has a good residuality on both mud and cement substrates with delayed mortalities above the WHO efficacy threshold for up to 11 months using the laboratory colony, and that it can control the wild colony for at least 8 months on cement substrates. Similar results were observed by Fongnikin et al. [22], who found that Fludora^®^ Fusion caused prolonged high mortality of pyrethroid-resistant malaria vectors for 7–10 months. Indeed, in our study, while a residual efficacy was observed at 2 months after 24 h exposure, the formulation failed to kill the mosquitoes at 8 months in the two supports at 24 h, confirming previous results obtained by Fuseini et al. [23] and Kamaraju et al. [9], respectively, on the island of Bioko and in Gurajat in India, where the residual efficacy of the same formulation was 7 months based on the estimates of 24 h mortality rates. However, the product was effective at 48 h, 72 h, and 96 h on both substrates, with higher residual efficacy on the cement substrate. The same effects were also observed for clothianidin on cement substrates and deltamethrin for both substrates with good efficacy at 48 h, 72 h, and 96 h. Thus, even if on the basis of the 24 h holding period, these observations reflect a better efficacy of Fludora^®^ Fusion compared to its constituents taken individually on cement substrates and to a lesser extent on mud substrates, the other two products were effective during the 8 months of monitoring with the resistant strain. The better effect observed on cement substrates could potentially be explained, as in several other studies, by a difference in texture between the two substrates, with the mud being more porous than the cement [8,12]. Indeed, it has now been shown that the nature of the substrate is an issue for the long-term efficacy of IRS [24,25,26]. Thus, with the rapid urbanization observed in several African contexts with the improvement of housing quality [27], with the use mainly of finished materials such as cement, IRS operations using Fludora^®^ Fusion have a better utility in Africa. However, it should be kept in mind that in the use of cone bioassays, mosquito exposure is forced. Indeed, previous studies in experimental huts have shown that free-flying mosquitoes in huts treated with the mixture are less affected by the treatments in comparison to clothianidin alone [22,28]. Therefore, our results using cone bioassays are likely to overestimate the performance of Fludora^®^ Fusion and deltamethrin compared to clothianidin alone. For the specific case of deltamethrin, which showed the residual efficacy on the local strain, the situation is questionable and requires further studies. As with the use of cone bioassays, the overestimation of induced mortality is possible with irritant insecticides such as deltamethrin, because even if mosquitoes do not land on treated surfaces, they may absorb more insecticide due to forced exposure in the cones.

## 5. Conclusions

This evaluation showed, under semi-natural conditions, a good efficacy and a residual effect of Fludora^®^ Fusion against laboratory and wild colonies. With this new formulation, the efficacy lasted at least 8 months and suggests its usefulness as an alternative product for vector control, as this observed period of efficacy covers the transmission period during the whole rainy season in most areas in Senegal.

## Figures and Tables

**Table 1:** Mortality rates of the laboratory and wild colonies to 0.05% deltamethrin.

Colonies	Tested	Alive	Dead	MR (%)
*An. coluzzii*	106	2	104	98.1 (93.3–99.8)
*An. arabiensis*	101	89	12	11.9 (6.3–19.8)

MR: mortality rate; ( ): 95% confidence interval.

## Data Availability

The data for this study have been presented within this article and any further information regarding this study can be reasonably requested from the corresponding author.
